# Supplementary material for: Population rate-coding predicts correctly that human sound localization depends on sound intensity
Source: eLife. 2019 Oct 21;8:e47027. doi: 10.7554/eLife.47027 (PMC6802950; doi:10.7554/eLife.47027)
Supplement: Transparent reporting form [file elife-47027-transrepform.pdf]

eLIFE
1st Floor
24 Hills Road
Cambridge CB2 1JP, UK
P 01223 855340
W elifesciences.org
T @elife

eLife's transparent reporting form

We encourage authors to provide detailed information *within their submission* to facilitate the interpretation and replication of experiments. Authors can upload supporting documentation to indicate the use of appropriate reporting guidelines for health-related research (see EQUATOR Network), life science research (see the BioSharing Information Resource), or the ARRIVE guidelines for reporting work involving animal research. Where applicable, authors should refer to any relevant reporting standards documents in this form.

If you have any questions, please consult our Journal Policies and/or contact us: editorial@elifesciences.org.

Sample-size estimation

- You should state whether an appropriate sample size was computed when the study was being designed
- You should state the statistical method of sample size computation and any required assumptions
- If no explicit power analysis was used, you should describe how you decided what sample (replicate) size (number) to use

Please outline where this information can be found within the submission (e.g., sections or figure legends), or explain why this information doesn't apply to your submission:

To estimate effect size, we initially performed pilot testing with three listeners. Bootstrap power analysis was performed on the pilot data for 300 repetitions. In each repetition, N listeners were sampled with replacement out of the total 3, and for each sampled listener 10 out of 20 observations of every ITD/SL pair were randomly selected. The resulting bootstrap data set was fitted with NLME and checked for significance of lateralization attributed to sound intensity (alpha = 0.05). Model fits that encountered convergence error were skipped (about 10% of repetitions, ~30 out of 300). Finally, the percentage of the repetitions that showed a significant effect of sound intensity were calculated and considered as statistical power of the data set.
Bootstrap analysis of pilot data shows a statistical power of 90% can be obtained with N=4 (95% with N=5, and 99% with N=10). Thus, we chose a sample size of N=10.

Replicates

- You should report how often each experiment was performed
- You should include a definition of biological versus technical replication
- The data obtained should be provided and sufficient information should be provided to indicate the number of independent biological and/or technical replicates
- If you encountered any outliers, you should describe how these were handled
- Criteria for exclusion/inclusion of data should be clearly stated
- High-throughput sequence data should be uploaded before submission, with a private link for reviewers provided (these are available from both GEO and ArrayExpress)

Please outline where this information can be found within the submission (e.g., sections or figure legends), or explain why this information doesn't apply to your submission:

Both behavioral experiments were performed once. Here, we define biological replicate as an individual listener and a technical replicate as an individual trial on which that listener was tested. Each biological replicate performed an identical number of technical replicates. The number of trials and statistical design are explicitly stated in Methods and Materials. Exclusion criteria, as described in detail in the manuscript, were as follows: listeners without normal hearing were excluded from training and testing (see criterion, lines 271-276). Unreliable reporters were excluded from testing (see criterion, lines 336-342). We encountered no outliers. No data was excluded.

eLife Sciences Publications, Ltd is a limited liability non-profit non-stock corporation incorporated in the State of Delaware, USA, with company number 5030732, and is registered in the UK with company number FC030576 and branch number BR015634 at the address 1st Floor, 24 Hills Road, Cambridge CB2 1JP | August 2014

Statistical reporting

- Statistical analysis methods should be described and justified
- Raw data should be presented in figures whenever informative to do so (typically when N per group is less than 10)
- For each experiment, you should identify the statistical tests used, exact values of N, definitions of center, methods of multiple test correction, and dispersion and precision measures (e.g., mean, median, SD, SEM, confidence intervals; and, for the major substantive results, a measure of effect size (e.g., Pearson's r, Cohen's d)
- Report exact p-values wherever possible alongside the summary statistics and 95% confidence intervals. These should be reported for all key questions and not only when the p-value is less than 0.05.

Please outline where this information can be found within the submission (e.g., sections or figure legends), or explain why this information doesn't apply to your submission:

We developed a non-linear mixed effects model, described in detail on page 11, lines 434-456. Figure 2 shows all data from behavioral testing, displaying both the raw data as well as the model fits. As stated explicitly in the text, error bars denote one standard error of the raw mean performance across listeners; error ribbons show one standard error of the model fits. All data from all listeners were modeled in the NLME statistical model. All exact model parameters, their SEMs, t-values and p-values are listed in Tables 1 and 2 as well as described in the text as appropriate.

(For large datasets, or papers with a very large number of statistical tests, you may upload a single table file with tests, Ns, etc., with reference to sections in the manuscript.)

Group allocation

- Indicate how samples were allocated into experimental groups (in the case of clinical studies, please specify allocation to treatment method); if randomization was used, please also state if restricted randomization was applied
- Indicate if masking was used during group allocation, data collection and/or data analysis

Please outline where this information can be found within the submission (e.g., sections or figure legends), or explain why this information doesn't apply to your submission:

This information does not apply to our submission, because we did not allocate our listeners into different experimental groups.

Additional data files ("source data")

- We encourage you to upload relevant additional data files, such as numerical data that are represented as a graph in a figure, or as a summary table
- Where provided, these should be in the most useful format, and they can be uploaded as "Source data" files linked to a main figure or table
- Include model definition files including the full list of parameters used
- Include code used for data analysis (e.g., R, MatLab)
- Avoid stating that data files are "available upon request"

Please indicate the figures or tables for which source data files have been provided:

All data, statistical analysis code (R), as well as our computational models (Matlab) are available at:
https://centers.njit.edu/nesh/neshbox/itdlevel/
